# Characterization and Simulation of the Bond Response of NSM FRP Reinforcement in Concrete

**DOI:** 10.3390/ma13071770

**Published:** 2020-04-09

**Authors:** Javier Gómez, Lluís Torres, Cristina Barris

**Affiliations:** AMADE, Polytechnic School, University of Girona, 17003 Girona, Spain; lluis.torres@udg.edu (L.T.); cristina.barris@udg.edu (C.B.)

**Keywords:** CFRP, NSM, bond behavior, structural behavior, material characterization, numerical modeling

## Abstract

The near-surface mounted (NSM) technique with fiber reinforced polymer (FRP) reinforcement as strengthening system for concrete structures has been broadly studied during the last years. The efficiency of the NSM FRP-to-concrete joint highly depends on the bond between both materials, which is characterized by a local bond–slip law. This paper studies the effect of the shape of the local bond–slip law and its parameters on the global response of the NSM FRP joint in terms of load capacity, effective bond length, slip, shear stress, and strain distribution along the bonded length, which are essential parameters on the strengthening design. A numerical procedure based on the finite difference method to solve the governing equations of the FRP-to-concrete joint is developed. Pull-out single shear specimens are tested in order to experimentally validate the numerical results. Finally, a parametric study is performed. The effect of the bond–shear strength slip at the bond strength, maximum slip, and friction branch on the parameters previously described is presented and discussed.

## 1. Introduction

The use of fiber reinforced polymer (FRP) materials for strengthening existing concrete structures has been widely studied during the last years because of the potential advantages of these materials compared to the conventional ones [1]. The most commonly used techniques for strengthening reinforced concrete (RC) structures with FRPs are the so-called externally bonded reinforcement (EBR) and near-surface mounted (NSM) reinforcement. While in the EBR methodology the FRP reinforcement is bonded on the exterior face of the structural element, in the NSM system the FRP is installed into groves cut in the concrete cover where it is bonded with the appropriate adhesive. The NSM technique has attracted the attention of researchers and industry in recent years due to several potential advantages compared to the EBR system, such as no need of specific surface preparation except grooving, better anchorage capacity, better protection in front of external agents like fire or vandalism, and no relevant change in the aesthetics of the structural element [2]. 

A number of research works have been carried out in order to obtain analytical solutions of the differential equations governing the bond performance of the FRP-to-concrete joint. This differential equation is based on the local bond–slip behavior, relating the bond and slip at every point along the bonded length. For the EBR technique, for instance, Yuan et al. proposed an analytical solution based on a local bilinear bond–slip law in [3] and a trilinear bond–slip one with an exponential descending branch in [4]. In a later work, Ali et al. [5] adapted Yuan’s analytical solution to the NSM strengthening system. Even though these authors proposed closed form equations to calculate the global bond behavior, these are not always easy to implement and are only suitable for specific local bond–slip laws.

In recent years, experimental and numerical studies have provided solutions to the global bond behavior of the EBR strengthening technique [6,7,8,9,10,11,12], although less attention has been paid to the NSM strengthening systems [13,14]. Focusing specifically on the characterization of the local bond behavior of NSM FRP-to-concrete joints, several bond–slip laws have been proposed in the literature, with different shapes and stages. Some examples are the models proposed by Sena et al. [13], Borchert et al. [14], Zhang et al. [15], and Seracino et al. [16]. The effect of their differences on the structural bond response and design implications is not always straightforward, thus, development of numerical tools that allow obtaining and comparing the response for different models is of main interest.

The aim of this paper is to study the effect of the shape of the local bond–slip law on the global bond behavior of NSM FRP strengthening systems. For this purpose, a numerical procedure based on finite difference method is proposed to solve in a general way the differential equation that characterizes the bond behavior of the FRP-to-concrete joint, which can be applied independently of the specific local bond–slip law. The numerical results are then verified by comparison between numerical and experimental results. Finally, a parametric study to evaluate the effects of the parameters defining the local behavior of the NSM FRP strengthened bonded joint is carried out.

## 2. Bond Mechanisms in NSM FRP Strengthening Systems

In the NSM FRP strengthening system, the forces are transferred from the FRP material to the internal faces of the concrete groove through its perimeter in contact with the adhesive. This way, there are two main issues improving the transfer of stresses with respect to the EBR reinforcement: the higher ratio of the area of the perimeter in contact with the adhesive to the FRP area, and the transmission to the concrete material through a confined zone in the interior of the slot in the concrete cover.

The global bond stress-slip response for NSM strips subjected to a pull-out force is generally characterized by an initial, relatively stiff, linear behavior, followed by a nonlinear curve up to the maximum value of the bond stress (bond strength), after which the originated damage causes a softening branch. Moreover, in NSM strengthening systems, it has been observed the activation of a friction component for relatively large slips as an extension of the softening branch [1,14,17,18,19]. The presence of a friction branch has been also reported in the bond behavior of other strengthening materials as fiber reinforced cementitious matrix composites (FRCM) [20,21].

The bond mechanisms generated in the NSM FRP technique can lead to several types of failure modes, which are mainly influenced by the bonded length, FRP surface and shape, groove configuration, materials mechanical properties and adhesive properties [22,23]. In general, the failure modes can be grouped into three main categories: failure at the FRP-adhesive interface, failure at the epoxy–concrete interface, and adhesive cover splitting [1]. 

The global bond stress-slip performance of an NSM FRP strip bonded to a concrete block is the result of the local bond–slip behavior at every point along the bonded length, which can be considered the constitutive model characterizing the bond behavior of the NSM FRP element. Different models and shapes can be found in the literature for these local laws, and the assessment of their results is the main objective of this paper.

## 3. Assessment of the Bond–Slip Response of NSM FRP

### 3.1. Governing Equation and Global Response

**Figure 1:**
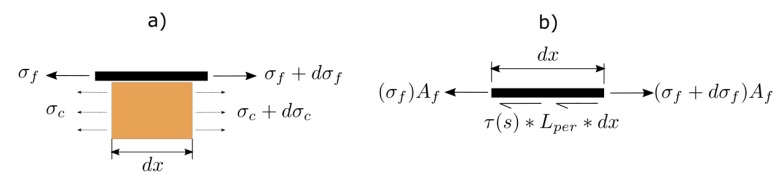
Stress equilibrium (**a**) in the FRP-concrete joint, and (**b**) in the FRP-adhesive interface.

In the single shear pull-out test (pull-push test), the load is transferred from the FRP to the concrete through the adhesive. Figure 1a shows the stress equilibrium of an infinitesimal element of FRP NSM concrete element of length *dx*. Figure 1b shows the stress equilibrium of the FRP-adhesive interface in an infinitesimal element of length *dx*.

From stress equilibrium between FRP and concrete, and the FRP-adhesive interface, Equation (1) and Equation (2) can be obtained.

(1) $$A_{f}d\sigma_{f}+A_{c}d\sigma_{c}=0,$$

(2) $$\frac{d\sigma_{f}}{dx}-\frac{\tau \left(s\right)*L_{per}}{A_{f}}=0,$$

where *σ_f_* is the stress in the FRP, *A_f_* is the FRP area, calculated as the product of the FRP thickness (*t_f_*) by the FRP width (*w_f_*), *σ_c_* is the stress in the concrete, *A_c_* is the area of concrete, *τ(s)* is the bond–slip law of the joint, and *L_per_* is the is the intermediate perimeter in the adhesive.

The slip of the bonded joint can be defined as the relative displacement between the FRP and the concrete element.

(3) $$s=u_{f}-u_{c},$$

where *s* is the slip, *u_f_* is the FRP displacement and *u_c_* is the concrete displacement. Using the constitutive equations of the FRP and the concrete, Equation (4) and Equation (5) are obtained.

(4) $$\frac{ds}{dx}=\varepsilon_{f}-\varepsilon_{c}=\frac{\sigma_{f}}{E_{f}}-\frac{\sigma_{c}}{E_{c}},$$

(5) $$\frac{d^{2}s}{dx^{2}}=\frac{1}{E_{f}}\frac{d\sigma_{f}}{dx}-\frac{1}{E_{c}}\frac{d\sigma_{c}}{dx},$$

where *E_f_* is the elastic modulus of the FRP, *ε_f_* is the strain in the FRP, *E_c_* is the elastic modulus of the concrete, and *ε_c_* is the strain in the concrete.

Finally, by substituting Equation (1) and Equation (2) into Equation (5), the differential equation governing the bond–slip behavior of a NSM FRP-to–concrete bonded joint is:

(6) $$\frac{d^{2}s\left(x\right)}{dx^{2}}-\tau \left(s\right)*L_{per}\left(\frac{1}{E_{f}*A_{f}}-\frac{1}{E_{c}*A_{c}}\right)=0,$$

It is worth mentioning that usually the second term in the parenthesis corresponding to the concrete properties is much lower than the first and can be neglected (Ko et al. [24]).

Equation (6) can be solved analytically using the boundary conditions

(7) $$\varepsilon_{f}=0\;\mathrm{at}\;x=0,$$

(8) $$s=s\left(L_{b}\right)\;\mathrm{at}\;x=L_{b},$$

where *ε_f_* is the strain in the FRP at the free end, *L_b_* is the bonded length, and *s*(*L_b_*) is the applied slip at the loaded end.

This differential equation can be solved for specific shapes of the local bond-stress slip law [3]. In the rest of cases a numerical procedure is required.

**Figure 2:**
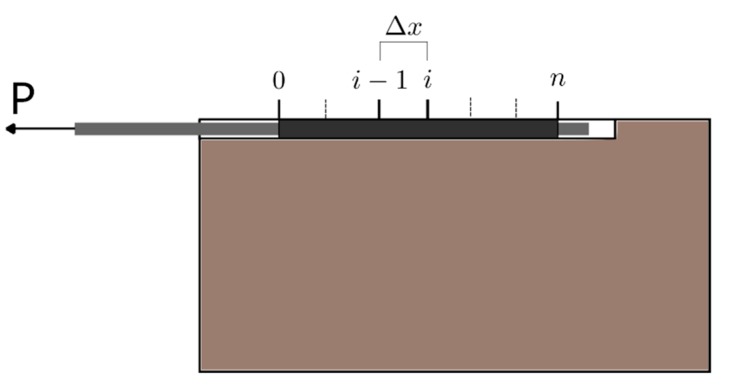
Scheme of the discretization along the FRP.

The numerical model implemented in this work is based on the finite difference method and aims to solve the governing equation of the bonded joint for any type of local bond–slip law. Following this methodology, the bonded length (*L_b_*) is discretized into *n* uniform small increments of length Δ*x = L_b_/n.* The increments are delimited by *n*+1 points, which position is defined by *x_i_* = *i*·Δ*x* (*i* = 0,1, …*n*) (Figure 2). The procedure is based on an incremental slip methodology that pretends to simulate a usual experimental test under monotonic increase of the loaded end slip (this way, possible snap-back effect after maximum load is not reproduced in the simulations, Zou et al. [21]). Starting from the loaded end and moving towards the free end, the procedure calculates, for every studied point, the slip and the load transmitted between two adjacent sections. For a certain value of slip at the loaded end, an iterative process is carried out in which *P(j,1)* is a lower bound value of the initial load at the loaded end at iteration *j*. *P(j,1)* is used to calculate the corresponding strain in the FRP (*ε_FRP_*), the strain in the concrete (*ε_c_*), and the bond–shear stress (*τ)* using the bond–slip law at the loaded point. Still, at iteration *j*, the load at every point *i*, *P*(*j,i*), is calculated using Equation (9) along the FRP and towards the free end, by

(9) $$P\left(j,i\right)=P\left(j,i-1\right)-\tau \left(j,i-1\right)*L_{per}*\Delta x,$$

From the load profile obtained using Equation (9), the strain distribution in the concrete and the FRP can be obtained, and, subsequently, the slip profile along the FRP is calculated as

(10) $$s\left(j,i\right)=s\left(j,i-1\right)-\left(\varepsilon_{f}-\varepsilon_{c}\right)*\Delta x,$$

**Figure 3:**
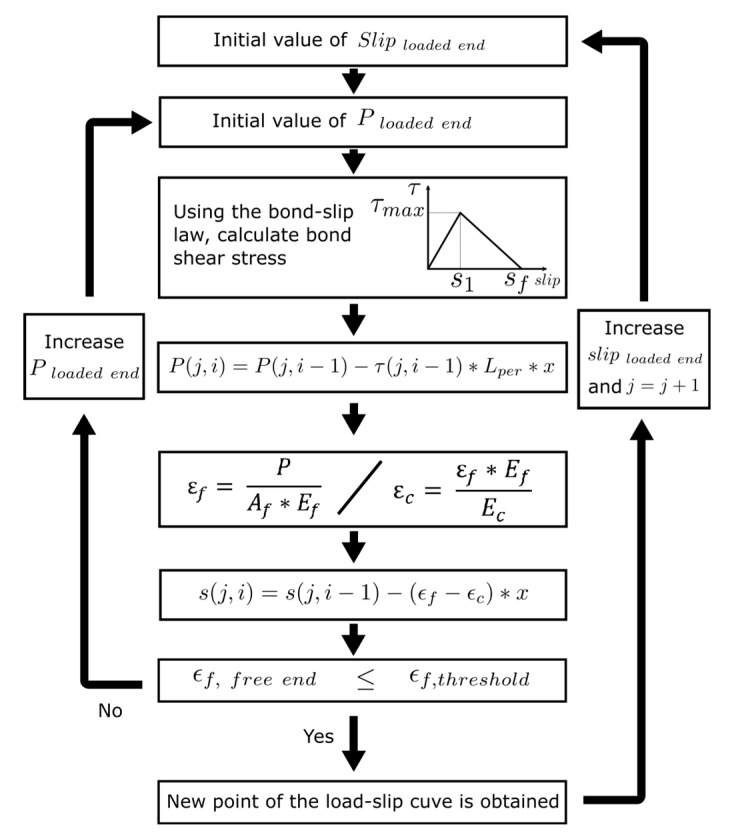
Flowchart of the numerical procedure.

Increments of *P* at the loaded end are applied until convergence is attained, which happens when the *ε_f_* at the free end is less than a prescribed tolerance, set to be a value close to zero. Once convergence is achieved, the corresponding load is registered and the process is repeated for a new value of slip at the loaded end, defined as *s(j+1,1)*. At this point, the iterative procedure is again repeated. The procedure finishes when all the points of the load–slip curve are calculated. Figure 3 shows the flowchart of the numerical procedure.

### 3.2. Comparison of the Numerical Results with Existing Analytical Solutions

**Figure 4:**
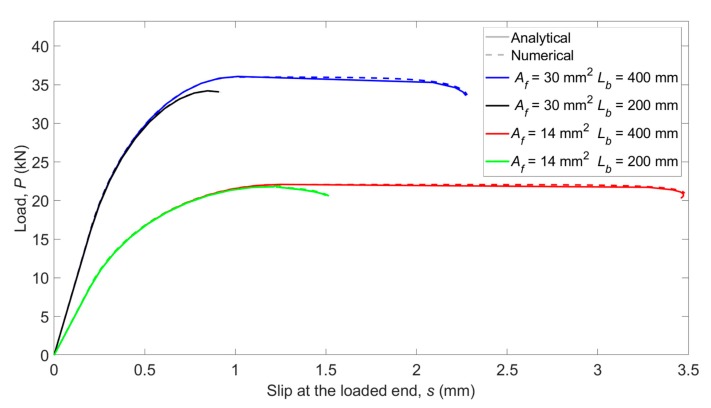
Comparison between the analytical and the numerical models.

In order to verify the numerical procedure developed, a comparison with analytical solutions available for some specific laws is presented in this section. For the sake of simplicity, only the analytical solution for the bilinear bond–slip law proposed by Yuan et al. [3] is taken as a reference, considering a bond–shear strength (*τ_max_*) of 15 MPa, a slip at the bond–shear strength (*s*_1_) of 0.1 mm and a maximum slip (*s_f_*) of 1.13 mm. These values have been chosen according to experimental results available in the literature. Four different situations are simulated by modifying the bonded length (*L_b_* = 200 and 400 mm) and the FRP area (*A_f_* = 14 and 30 mm^2^). The results, presented in Figure 4, show good agreement between the analytical and the numerical solutions, and therefore the suitability of the developed procedure. 

## 4. Bond–Slip Behavior at a Local Level

In this work, six different models (Table 1) based on two of the most widely accepted constitutive laws for bond characterization of NSM FRP systems (the Bilinear law [16] and Borchert law [14]) are considered. The study focuses on the effect of the combination of their parameters on the structural global response of the bonded joint. From the bilinear law, four combinations are studied: i) a bilinear (BL) model; ii) a linear descending (LD) model, which does not consider the initial elastic branch; iii) a two stage bond–slip law with a non-linear ascending branch (TSANL) model, with the aim to study the effect of the shape of the ascending branch; and iv) a bilinear plus friction (BLF) model, with the purpose to obtain the effect of friction in a bilinear bond–slip law. On the other hand, from Borchert law (BO), one additional modification is proposed, consisting in suppressing the original bond–shear strength plateau (BONP). 

## 5. Global Bond–Slip Response for Different Local Bond–Slip Laws

The main objective of this section is to evaluate the influence of the local bond law on the global behavior of the FRP laminates bonded to concrete as NSM strengthening system, when subject to a pull-out force, using the numerical model described in Section 3, and the bond–slip laws introduced in Section 2.

### 5.1. Parameters Used

The values of the parameters that define the different local bond–slip laws, as well as the properties of materials and characteristics of specimens have been chosen according to previous experimental campaigns found in the literature [13,14,15,16,25,26], with the aim to simulate conditions as realistically as possible.

The elastic modulus of the laminate and concrete are 150 GPa and 33 GPa, respectively. The concrete block cross-section is 200 × 200 mm and the groove thickness and width are 5 mm and 15 mm, respectively. The bonded length (*L_b_*) has been set as 200 mm, and the laminate section is 1.4 × 10 mm.

Regarding the bond–slip parameters, bond–shear strength has been set as 15 MPa, and the corresponding slip as 0.1 mm. In bond–slip models that have a friction branch, *τ_f_* has been defined as the 35% of *τ_max_*. The initial point of the plateau of BO model is $s_{1}=0.8\cdot s_{2}$, and the experimental parameter *α* is 0.31 [14].

**Figure 5:**
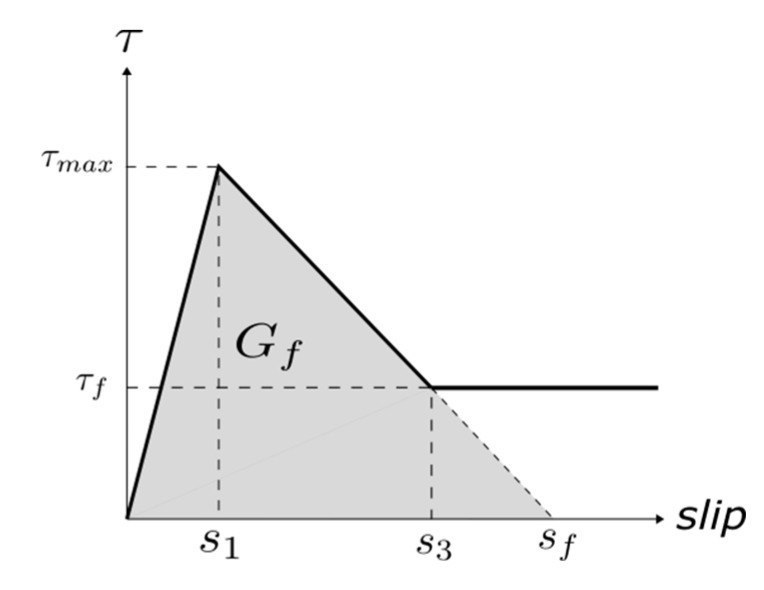
Fracture energy obtained from the bond–slip law.

Another parameter that is crucial for the simulations is the fracture energy (*G_f_*), typically defined as the area under the bond–slip law [6]. In those bond–slip laws with a friction branch, *G_f_* is calculated according to Haskett et al. [27], as the area under the ascending and descending stages of the bond–slip law, without taking into account the friction stage, as illustrated in Figure 5. A value of 8.5 N/mm has been set for *G_f_* in all the models [16,25,26].

**Figure 6:**
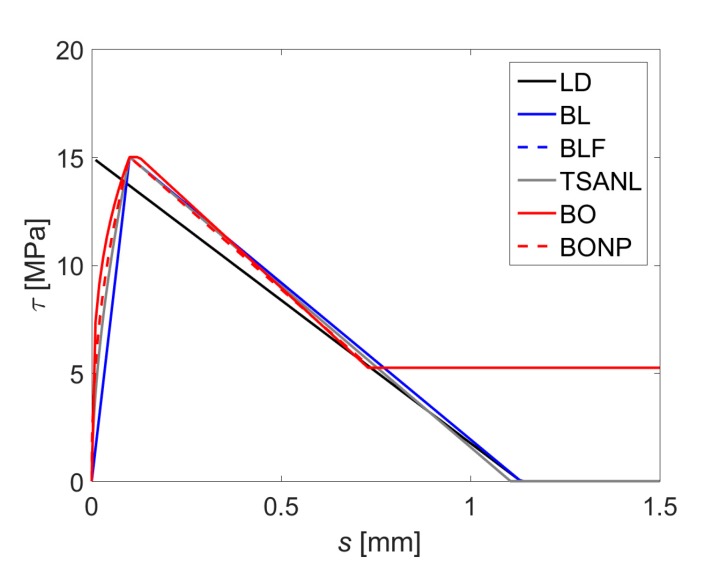
Comparison of the bond–slip models.

The comparison between the studied bond–slip laws is shown in Figure 6. As indicated before, it can be observed that the bond–shear strength (*τ_max_*) is 15 MPa, and the corresponding slip (*s*_1_) is 0.1 mm, except for the LD model, which does not consider *s*_1_. 

### 5.2. Load–Slip Response

**Figure 7:**
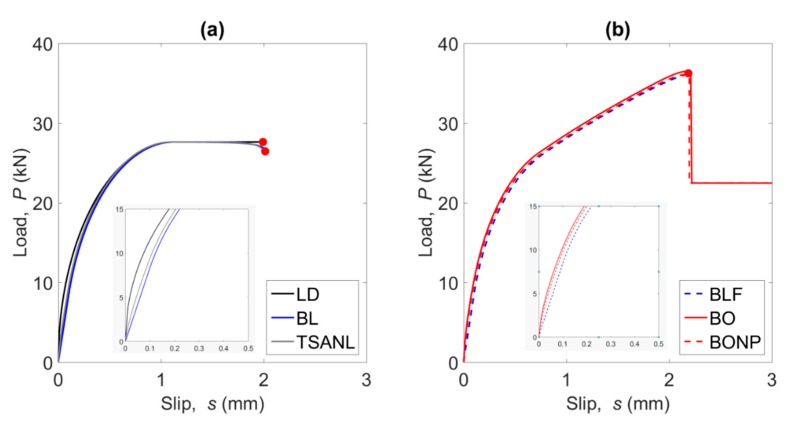
Comparison of the load–slip curves, (**a**) shows LD, BL and TSANL models, and (**b**) shows BLF, BO, and BONP models.

The comparison of the load–slip curves obtained from each bond–slip law is shown in Figure 7. Overall, two main tendencies can be observed. The first one (Figure 7a), followed by LD, BL and TSANL models, achieve a maximum load, which remains constant as the slip in the loaded end increases until failure. This plateau is obtained because the maximum activated length is attained when the slip in the loaded point is equal to *s_f_*, meaning that this point does not carry load anymore (debonding). Therefore, the load capacity will remain constant and the slip will keep increasing until failure. In the second trend (Figure 7b), followed by BLF, BO, and BONP models, the load increases up to the maximum carrying capacity, where a sudden decrease takes place followed by a residual (friction) bond force. The difference between these two trends falls to the effect of the friction branch of the local bond–slip law after the loaded point reaches debonding. 

Focusing on the initial part of the load–slip curve (interior plot in Figure 7a) it can be seen that the LD model shows the highest stiffness, because the ascending branch of the bond–slip law has an infinite stiffness. Besides, it can be observed that bond–slip models with a non-linear ascending branch (TSANL, BO, and BONP) exhibit a stiffer branch of the load–slip curve than BL and BLF models caused by the fact that for a fixed value of *s*_1_ the area under the ascending branch is greater in those cases with non-linear tendency.

### 5.3. FRP Strain, Bond–Shear Stress and Slip along the Bonded Length

**Figure 8:**
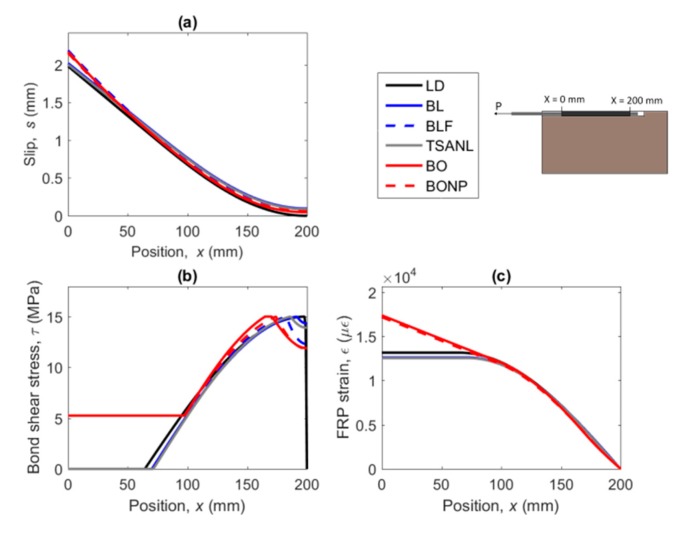
(**a**) FRP slip, (**b**) bond–shear stress, and (**c**) FRP strains along the bonded length for the different bond–slip models.

Following the numerical procedure, the FRP strain, bond–shear stress and slip can also be obtained. Figure 8 shows the response along the bonded length for the different laws (loaded end at *x* = 0 mm, free end at *x* = 200 mm) evaluated at the situation of imminent failure (marked with a red dot in Figure 7). For 7.5 mm grooved specimens, the failure point was defined as the point before the load drops to 0, and for 10 mm grooved specimens, the failure was defined as the maximum load point.

From Figure 8a it can be observed that the shape of the bond–slip law has a small effect on the slip profile along the FRP. It can also be seen that BLF, BO and BONP exhibit slightly higher values for the slip at the loaded end. Furthermore, the slip at the free end equals to 0 for the LD model, contrarily to the rest of models, which show a small value of slip.

An evident difference between the models with and without friction can be observed in the bond stress distribution along the bonded length (Figure 8b). Models that include friction still transmit bond–shear stress after the softening stage, whilst the other models exhibit a completely damaged zone, where no load is transmitted. Hence, for friction bond–slip models, the increase of the area under the bond–shear stress profile allows the joint to carry more load even though the zone near the loaded end is damaged. 

Figure 8c shows the FRP strain distribution. In models without friction, the strain profile stabilizes when the maximum load is attained. On the other hand, strain values in the FRP do not stabilize for BLF, BO, and BONP models, because of the load keeps increasing after the loaded end surpasses the softening stage.

## 6. Experimental Program

In this section, an experimental program is presented and the results, in terms of load–slip curves are discussed. Then, a relatively simple methodology to obtain the bond–slip law from the experimental load–slip curve is presented. Finally, the theoretical results obtained from the numerical procedure are compared to experimental values.

### 6.1. Material Properties

The concrete used in the experimental campaign has been characterized according to the UNE 12390-3 [28] and the ASTM C469 / C469M-10 standard [29]. From the characterization tests on 150 × 300 mm cylindrical specimens, a compressive strength of 36.9 MPa and an elastic modulus of 46.8 GPa were obtained. 

Carbon-FRP (CFRP) strips with 3 × 10 mm cross-section were used in the experimental campaign. Their mechanical properties were tested according to ISO 527-5 [30], obtaining an elastic modulus of 169.3 GPa and a tensile strength of 3205.9 MPa. 

The bi-component epoxy resin used in this program was tested according to the ISO 527-2 standard [31] after 12 days of curing under 20ºC and 55%RH, obtaining an elastic modulus of 10.7 GPa and a tensile strength of 27.9 MPa.

### 6.2. Experimental Details

#### 6.2.1. Parameters of the Study 

Four different NSM configurations were tested, combining two different groove thicknesses (7.5 mm and 10 mm) and two different bonded lengths (150 mm and 225 mm). Four specimens were tested per each configuration, giving a total of 16 specimens tested in a direct pull-out shear test.

The *fib* Bulletin 90 [1] limits the deepness of the groove in order to avoid epoxy cover splitting. ACI [32], in turn, suggests that the groove thickness should be, at least, 3 times the laminate thickness. In order to study the effect of the resin layer thickness, two groove thicknesses were defined in this study: 10 mm and 7.5 mm. The first one was set according to the ACI requirement, whilst the second groove thickness was chosen to satisfy the *fib* [1] condition that establishes that the minimum groove dimensions must be 1.5 or 2 times the laminate size. 

Regarding the effect of the bonded length, Seracino et al. [33] used bonded lengths between 100 mm and 350 mm and concluded that the minimum *L_b_* to achieve the maximum carrying capacity was 200 mm. Furthermore, Zhang et al. [34] tested NSM strengthened concrete elements with bonded lengths between 25 and 350 mm. From their study, it was observed that the experimental effective bonded lengths for the different specimens were between 150 and 175 mm. 

The bonded lengths used in this experimental campaign were chosen to be above and below the values suggested in [33] and [34], in order to validate if the finite differences model is able to predict the response for short and long bonded lengths. 

#### 6.2.2. Test Setup 

In order to study the effect of the bonded length and the groove width, four pull-out configurations were designed. Four specimens were tested for each configuration defined in Table 2.

**Figure 9:**
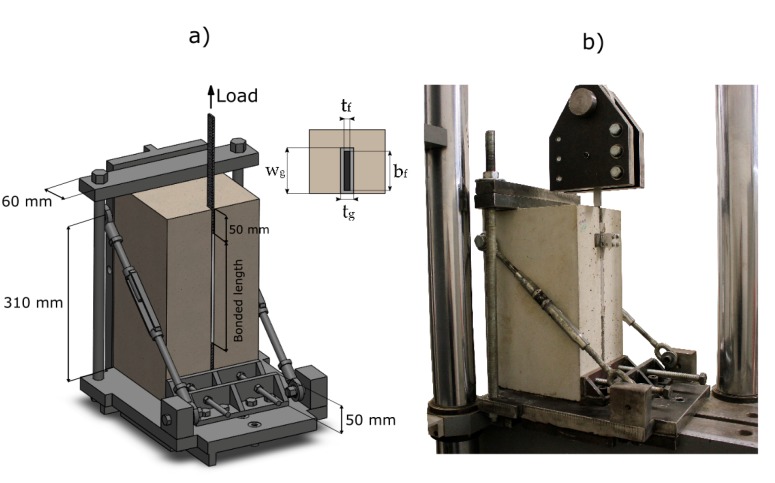
(**a**) scheme of the set-up for the pull-out single shear test, and (**b**) picture of the tests.

The specimens were identified as NSM – *L_b_* – *t_g_*, where *L_b_* stands for the bonded length and *t_g_* for the groove thickness. The tests were carried out under a single shear test configuration, as shown in Figure 9**.** The specimen was rigidly fixed with a 60 mm wide plate to avoid translation in the vertical direction and also perpendicularly in the opposite side with a 50 mm plate to avoid rotation. At the top of the specimen a length of 50 mm was left unbonded to avoid stress concentrations in that zone [35]. The concrete block dimensions used on the short-bonded length specimens were 200 × 200 × 370 mm, and for the long-bonded length specimens 200 × 200 × 420 mm. A servo-hydraulic testing machine was used to apply the load. The test was performed under displacement control with a speed of 0.2 mm/min. One LVDT was placed in the loaded end to measure the relative displacement between the CFRP strip and the concrete. 

### 6.3. Experimental Results

**Figure 10:**
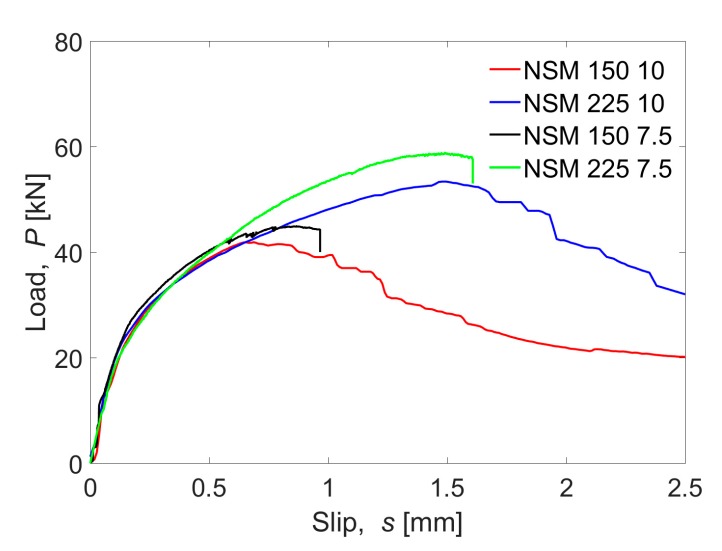
Experimental results from the single shear tests.

The results obtained from the experimental tests are shown in Figure 10, where the average load–slip curves for the four tested configurations are presented.

Failure loads for the narrowest groove specimens were slightly higher than for those with the widest grooves: the failure load of NSM-150-7.5 was 7.1% higher than NSM-150-10, and NSM-225-7.5 failure load was 10.0% higher than in the NSM-225-10 case. Besides, differences in the failure load can be observed: 10 mm grove thickness specimens failed in the FRP-adhesive interface while 7.5 mm grove thickness specimens failed in the resin–concrete interface. 

As can be observed, in specimens with 10 mm groove thickness, after reaching the maximum load, the slip continued increasing although the load decreased, which could be interpreted as all the bonded length being activated and damaged, as well as friction effect taking place. On the other hand, failure of the specimens with 7.5 mm groove was sudden and instantaneous: after reaching the maximum load, the load capacity dropped abruptly with no friction effects. For this reason, in this study, two different bond–slip laws have been defined: a bilineal law for the case of 7.5 mm groove specimens and a bilineal plus friction law for the case of 10 mm groove specimens. Finally, it should be noticed that the slip at the end of the elastic stage and at the maximum load was similar both for specimens with 10 mm and 7.5 mm groove thickness.

Regarding the effect of the bonded length, it can be seen that the initial tendency of the load–slip curves was the same for both bonded lengths. On the other hand, as the bonded length increased, the maximum load carrying capacity increased as well. Increasing the bonded length 50% caused a load increase of 24.8% and a 22.4% for specimens with 10 mm and 7.5 mm groove thickness, respectively. 

### 6.4. Bond–Slip Law Adjusted to the Experimental Results

**Figure 11:**
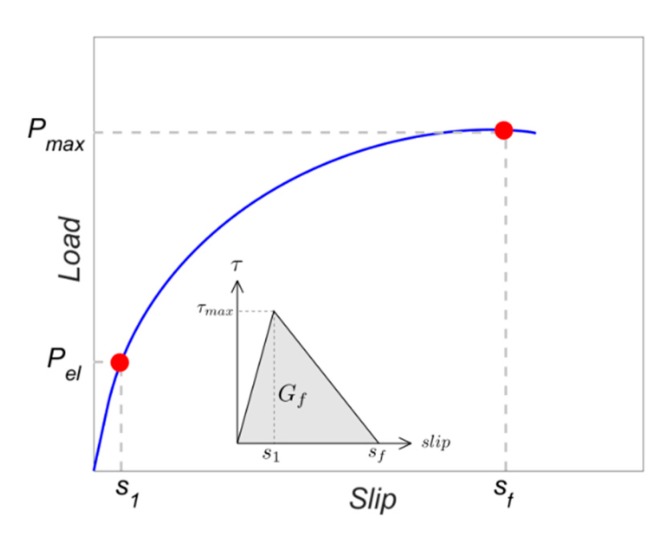
Bond–slip law parameters obtained from the load–slip curve for 7.5 mm grooved specimens.

From the experimental tests results, a bilinear bond–slip law could be obtained from the load–slip curves [36] for specimens with 7.5 mm groove and 10 mm groove. For the case of specimens with 7.5 mm groove thickness, the slip at the bond strength (*s*_1_) was obtained at the end of the linear branch of the load–slip, whilst the maximum slip (*s_f_*) could be estimated from the slip where the maximum load was achieved (Figure 11). Once *s*_1_ and *s_f_* were estimated, the value of the bond–shear strength (*τ_max_*) was obtained from a least-squares approach that estimated the optimum value of *τ_max_* to obtain the experimental load until *P_max_*.

For specimens with 10 mm groove thickness, which present a frictional stage after the maximum load is achieved, a trilinear bond–slip model with a friction branch has been adopted. In this model, *s*_1_ and *s_f_* are assumed equal to the values obtained for the 7.5 mm grooved specimens. Once the values of *s*_1_ and *s_f_* are defined, the methodology to calculate *τ_max_* is the same as that defined for 7.5 mm grooved specimens. The friction bond–shear strength (*τ*_f_) was defined as 35% of τ_max_, following the reccomendations in [14].

**Figure 12:**
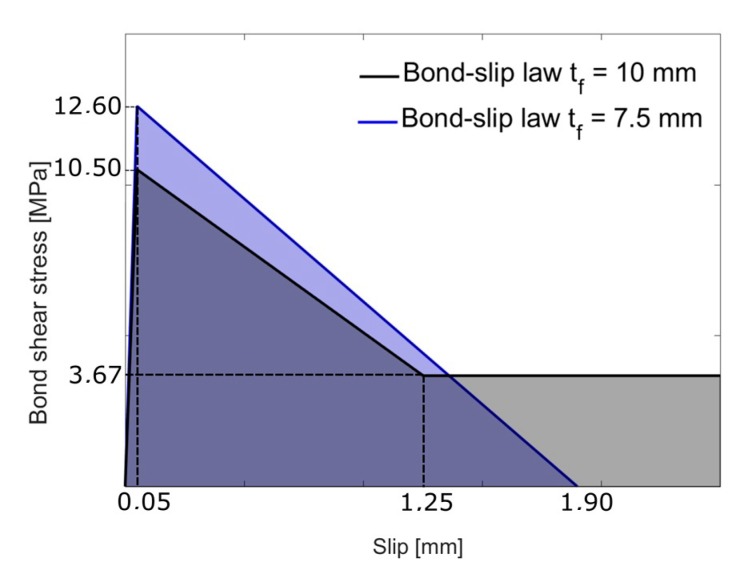
Experimental bond–slip laws for the NSM with 7.5 mm and 10 mm thickness grooves.

Figure 12 shows the two adjusted bond–slip laws, for 7.5 mm and for 10 mm groove specimens, with their corresponding parameters.

It can be observed that for the specimens with 7.5 mm groove thickness, *τ_max_* is higher than for specimens with 10 mm groove thickness, implying higher fracture energy and justifies the higher maximum load experimentally obtained. 

### 6.5. Comparison between Experimental and Numerical Results

**Figure 13:**
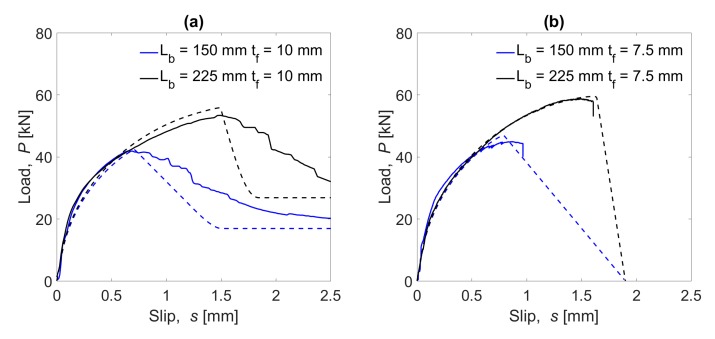
Comparison between the experimental (continuous lines) and the theoretical (dashed lines) values for the NSM with **(a)** 10 mm and **(b)** 7.5 mm groove thickness.

By introducing the previously obtained bond–slip laws and the materials’ mechanical properties in the finite differences model, the theoretical load–slip curves of the bonded joints are obtained. The comparison between the theoretical and experimental behavior is shown in Figure 13.

As expected, a close agreement between the predicted response and the experimental values is observed. Focusing on Figure 13a (10 mm groove thickness), it can be seen that the ascending stages of the load–slip curves and the maximum loads are correctly predicted, although the experimental results show a smoother decrease of the load once the maximum load is attained than the evolution predicted by the theoretical model. In the case of Figure 13b (7.5 mm groove thickness), the ascending branch and the maximum load are also correctly predicted. 

## 7. Parametric Study

In the previous section, the bilinear and bilinear + friction bond–slip laws were implemented in the finite differences model. In this section, a parametric study is performed using the six different bond–slip models defined in Section 2 (Table 1) to better investigate the influence of other modifications of the bond–slip law.

The effect of four bond–slip law parameters on the maximum load (*P_max_*) and on the effective bonded length (*L*_eff_) are studied. The studied parameters are: i) the bond–shear strength (*τ_max_*), ii) the slip at the bond–shear strength (*s*_1_), iii) the maximum slip (*s_f_*) and iv) the friction bond–shear strength (*τ*_f_). *L*_eff_ is defined here as the bonded length needed to withstand the maximum stabilized load, therefore, it is only applicable to bond–slip models without friction branch. Computationally, the calculation of *L*_eff_ is measured from the first point that achieves *s_f_* until the point that has less than 3% of the maximum FRP strain [5]. 

### 7.1. Effect of the Bond–Shear Strength (τ_max_)

**Figure 14:**
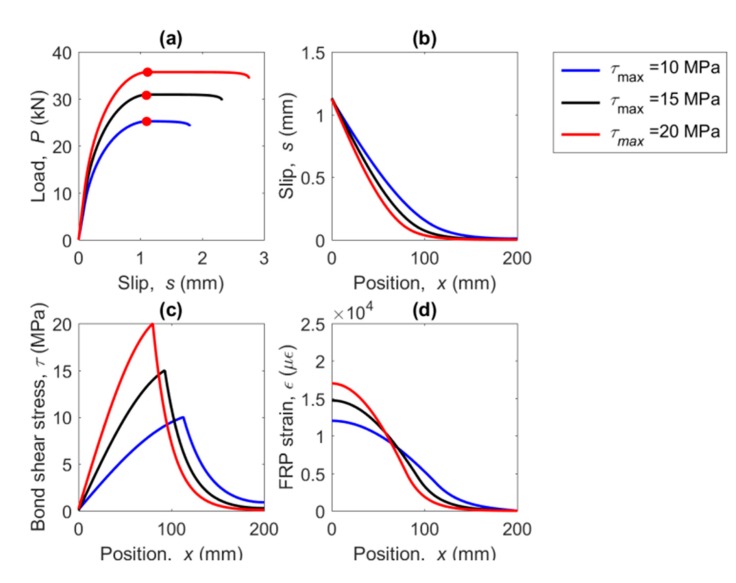
(**a**) Load–slip curve, (**b**) slip along the FRP, (**c**) bond–shear stress along the FRP, and (**d**) strains along the FRP for three values of bond–shear strength and a bilinear bond–slip law.

Using the numerical model, the load–slip curve, and the slip, bond–shear stress and FRP strain profile along the bonded length are obtained. For the sake of brevity, only the response of the BL model is shown in Figure 14, however, the trends observed for the BL model can be extrapolated to the other bond–slip models. A range of *τ_max_* between 10 MPa and 20 MPa has been considered taking *τ_max,_*_0_ = 10 MPa as a reference for normalization of results and, being *s_f_* = 1.13 mm, *s*_1_ = 0.1 mm, *s*_2_ = 0.12 mm, and *τ_f_* = 5.25 MPa.

In Figure 14, the curves are represented for the situation where the slip at the loaded end arrives at *s_f_*. It is clearly seen that *P_max_* increases with *τ_max_* (Figure 14a). This is because an increase of *τ_max_* implies an increase of *G_f_*, thus, a higher load is needed to damage the bonded joint. In Figure 14b, where the slip profile is shown, it can be observed that as the *τ_max_* increases, the activated bonded length decreases. At *x* = 200 mm the slip in the free end when *τ_max_* is 10 MPa, is equal to 0.0091 mm, but when *τ_max_* is 20 MPa, the slip in the free end is negligible, meaning that the activated length decreases with the increase of *τ_max_*.

In Figure 14c the bond stress profile is represented. In that case, it can be seen that as *τ_max_* increases, the bond–shear profile becomes narrower because every segment along the bonded length is able to transfer a larger bond–shear force. Finally, as the bond–shear strength (*τ_max_*) increases, the strain in the loaded end increases as well (Figure 14d). It is worth noticing that as the bond–shear strength capacity increases, the activated bonded length decreases.

**Figure 15:**
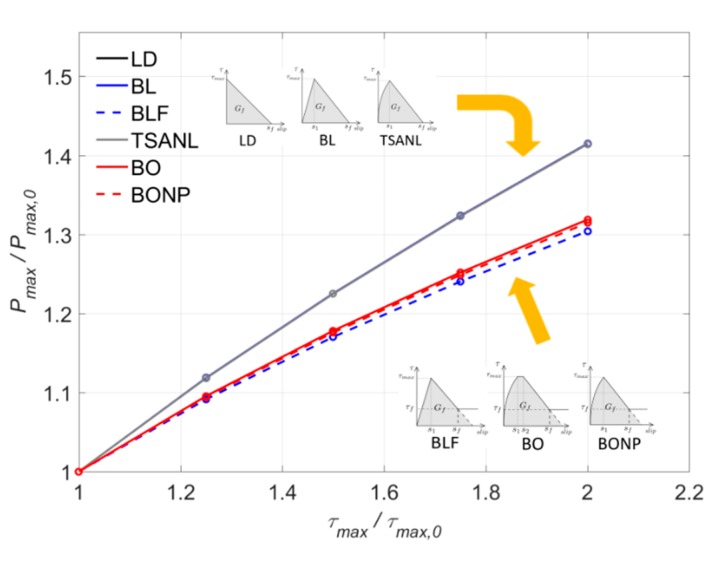
Variation of P_max_ versus τ_max_.

In Figure 15, the increase of the maximum load is depicted in function of the increase of the *τ_max_*, previously normalized with respect to *τ_max,_*_0_ = 10 MPa. In general terms, a practically linear increase of the maximum load with the bond–shear strength can be observed for all the models. This is due to the fact that increasing *τ_max_* without changing the value of *s_f_* causes a linear increase of the fracture energy (*G_f_*), leading to a situation where a higher load is needed to damage the bonded joint.

Moreover, it can be seen that LD, BL, and TSANL models show very similar results, meaning that these models are analogously affected by the increase of the bond–shear strength. These models show an increase of *P_max_* of 41% when *τ_max_* is 20 MPa. Finally, BLF, BO, and BONP, show lesser effect on the maximum load than the other models, because of the friction branch, which remains constant for all the cases. Although these models increase their *G_f_*, the increase of the area under the bond–slip curve is smaller than in the non-friction cases. The increase of *P_max_* in these models arrives to 30% when *τ_max_* is 20 MPa. Finally, the BLF model is slightly less affected than BO and BONP models, because of the non-linear ascending branch of Borchert models, which experiment a higher increase of *G_f_* when *τ_max_* increases. 

**Figure 16:**
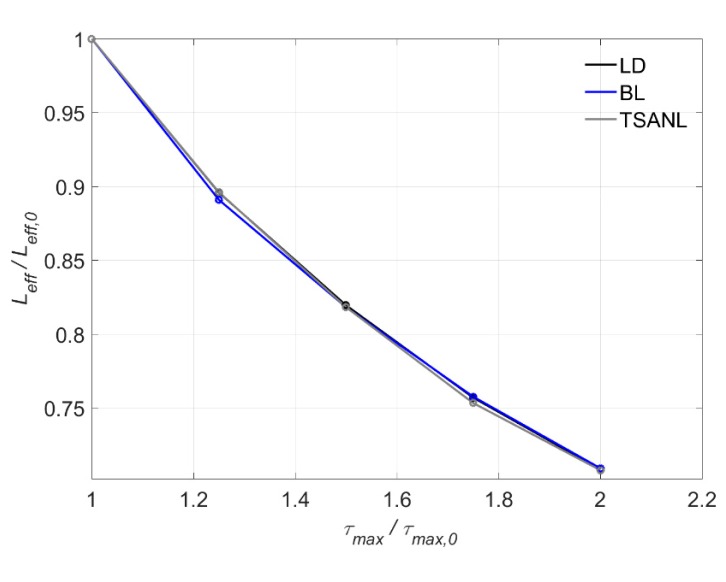
Variation of L_eff_ versus τ_max_.

The influence of *τ_max_* on the effective length, *L*_eff_, is shown in Figure 16 for models without friction. In general, a decrease of its value is observed as *τ_max_* increases, as expected: if the bond–shear strength increases, every finite element of the bonded length can transfer a larger shear force, hence, less bonded length is needed to transfer the total load. It is worth noticing that the three models behave in a very similar way, indicating that assuming either the one stage model, the bilinear model or the model with a non-linear ascending stage, does not provide a difference on the effective bonded length for different bond strengths.

### 7.2. Effect of the Slip at the Bond–Shear Strength (s_1_)

A range for *s*_1_ between 0.1 mm and 0.5 mm has been considered, taking *s*_1,0_ = 0.1 mm for normalization of results, and keeping *τ_max_* = 15 MPa. The value of the *G_f_* when *s*_1,0_ = 0.1 mm is equal to 8.5 N/mm. In order to meet the new value of *s*_1_, *G_f_* varies between 8.5 N/mm and 10.9 N/mm, depending on the model considered. In BO model, the difference between *s*_1_ and *s*_2_ has been kept constant for all cases. The linear descending (LD) model is not included because the slip at the bond–shear strength is 0, and if this point is shifted horizontally, it would become a bilinear (BL) model.

**Figure 17:**
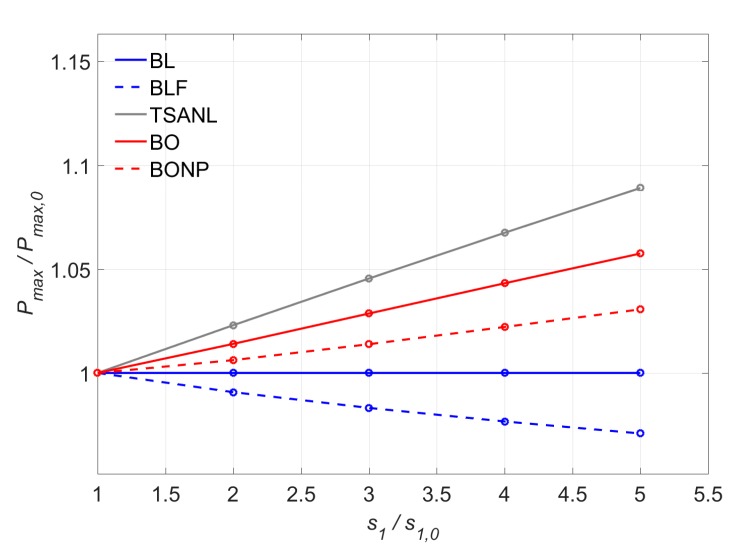
Variation of P_max_ versus s_1_.

The results are plotted in Figure 17 in terms of increment of the maximum load with respect to the increment of *s*_1_. As observed, in all models the load increment is considerably small (with a maximum variation of 8.9%). For the BL model, no effect on the maximum load is obtained for a substantial increment of *s*_1_, because *τ_max_* and *s_f_* are constant, and therefore, the fracture energy (*G_f_)* remains the same.

In the case of the BLF model, the maximum load decreases as the *s*_1_ increases. Even though the fracture energy (*G_f_*) remains constant for all the *s*_1_ values, as can be seen in Table 3, the area under the total bond shear–slip curve, considering the three stages (elastic, softening and friction) sligthly decreases with the increment of *s*_1_. It should be noticed, however, that the maximum load decrease only arrives up to 3% of the initial load.

It is also seen that bond–slip models that have a non-linear ascending branch (TSANL, BO, and BONP models) exhibit an some increase on the maximum load (*P_max_*), caused by the increase of area under the elastic stage curve from the shifting of *s*_1._ This effect can be observed in Table 3 through an increment of the fracture energy (*G_f_*) with the increase of *s*_1_. It should be noticed that the variation of the fracture energy for TSANL and BONP models is the same, therefore, in Table 3, only BONP model is showed. In BO and BONP cases, the increase of the fracture energy is mitigated by the decreasing of friction area between *s*_3_ and *s_f_* (Table 1): since the value of *s_f_* is kept constant and *s*_1_ increases, the value of *s*_3_ shifts horizontally, causing a reduction of area between *s*_3_ and *s_f_*.

The TSANL model is more affected by the modification of *s*_1_ than the other models, since it does not have a friction branch and the total area under the bond–slip law will not decrease with the shifting of *s*_1_, and the increase of the fracture energy will not be mitigated. The increase of *G_f_* in the TSANL model is the same as in the BONP model, since the definition of *G_f_* has been considered as in Figure 5.

**Figure 18:**
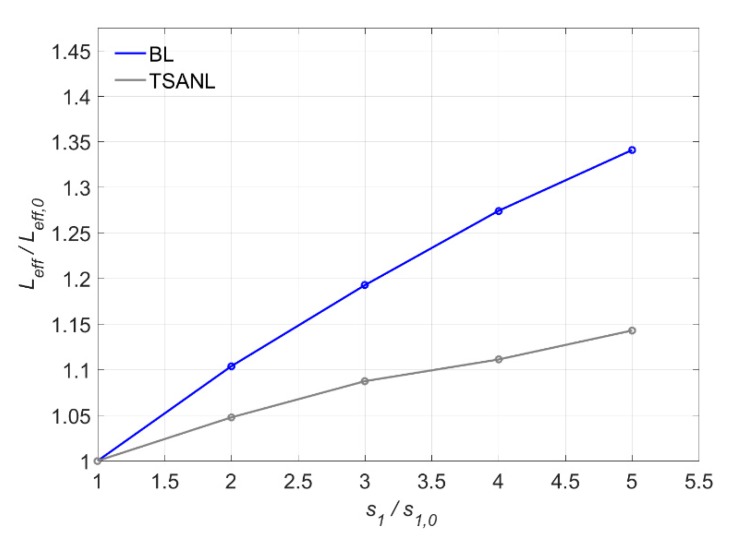
Variation of Leff versus s_1_.

Figure 18 shows the variation of the effective bonded length with *s*_1_ for TSANL and BL models. The effective bonded length (*L*_eff_) increases as *s*_1_ increases. Since higher slips are obtained in the elastic stage, a longer bonded length will be activated and *L*_eff_ will increase.

Moreover, the BL model is more sensitive to the variation of *s*_1_ than the TSANL model, because the change in the non-linear ascending branch of the TSANL model slightly increases the area under the elastic branch, improving the resistance of the joint under in this stage. This way, the increase of slip caused by the *s*_1_ shifting is mitigated by this increase of resistance.

### 7.3. Effect of the Maximum Slip (s_f_)

**Figure 19:**
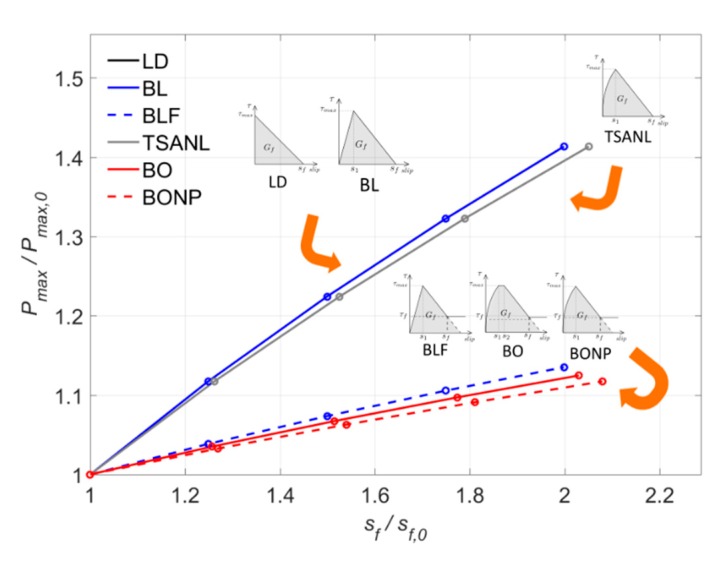
Variation of P_max_ versus s_f_.

Fixing the values of the bond–shear strength (*τ_max_*) and the slip at the b ond shear strength (*s*_1_) to 15 MPa and 0.1 mm, respectively, and setting the fracture energy (*G_f_*) to the values obtained in Section 7.1 (ranging between 8.5 N/mm and 11.33 N/mm) for each model, the values of the initial maximum slip (*s_f_*_,0_) are between 0.70 mm and 0.75 mm. Because the bond–slip models have different shapes, and therefore, different values of *G_f_*, the maximum slip, which satisfies the conditions of *τ_max_* and *s*_1_, will be different for each bond–slip law, as seen in Figure 19.

Overall, in Figure 19 it can be seen that all the models show an increasing tendency of *P_max_* with *s_f_*, because as *s_f_* increases, *G_f_* increases as well. The LD model presents exactly the same curve as the BL, since in both cases *G_f_* is the same. Moreover, as indicated in previous subsections, models without friction branch—such as LD, BL, and TSANL—are more sensitive to the increase of *s_f_* because the shifting of the slip affects directly to *G_f_, P_max_* increases up to 41% when *s_f_* is the highest value. It is worth noticing that the TSANL is slightly less sensitive because the area under the elastic branch is higher than in LD and BL models, causing that the increment of *s_f_* affects proportionally less to *G_f_* and *P_max_*, around 13% when *s_f_* is the maximum value. As for the models with friction branch—BLF, BO, and BONP—increase the *P_max_* caused by the shifting of *s_f_* is diminished by the effect of the friction branch in the total area as indicated in previous subsections. 

**Figure 20:**
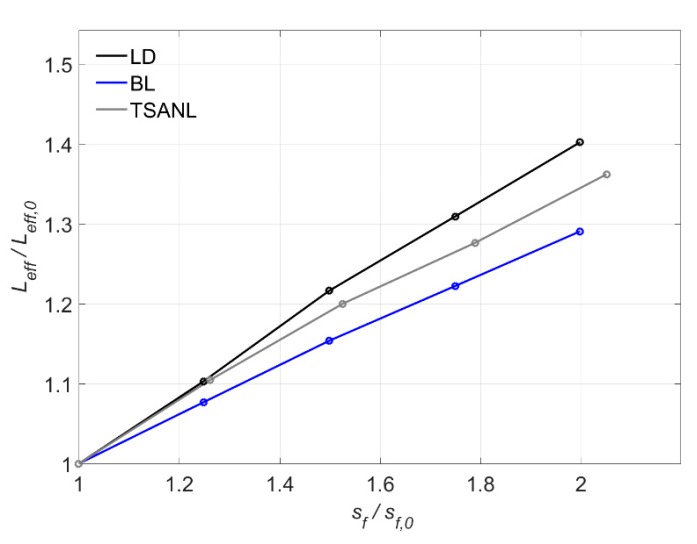
Variation of *L*_eff_ versus *s_f_*.

Figure 20 shows that *L_eff_* increases with the increase of *s_f_* for all models. A higher *s_f_* allows the bonded joint to have higher slips while transferring load, and consequently, more bonded length is activated. LD model exhibits the highest influence of *s_f_* and BL model is the less affected model. 

### 7.4. Effect of the Friction Branch (τ_f_)

Only BLF, BO, and BONP (with friction branch) models are included in this section. The bond–slip law parameters are set to *τ_max_* = 15 MPa, *s*_1_ = 0.1 mm, and *G_f_* = 8.5 N/mm. The bond–shear stress of the friction branch ranges between 10% and 40% of *τ_max_*, therefore *τ*_f_ will vary between 1.5 MPa (*τ*_f,0_) and 6 MPa.

**Figure 21:**
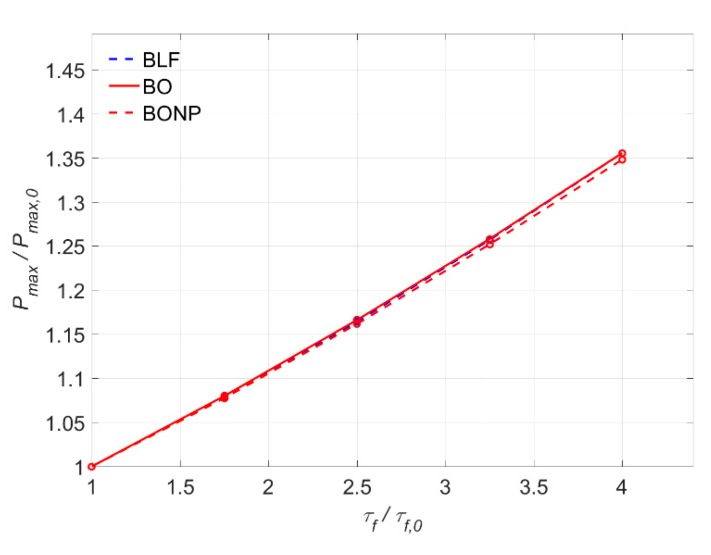
Variation of P_max_ versus τ_f_.

Figure 21 shows the variation of *P_max_* with the increase of *τ*_f_. It can be seen that all models present the same almost linear rising tendency, as expected. For example, for an increase of 2.5 times *τ*_f,0_ (i.e., *τ*_f_ = 3 MPa), the increase of *P_max_* is around 17%, while for 4 times *τ*_f,0_ (*τ*_f_ = 6 MPa), *P_max_* increases around 35%. 

## 8. Conclusions

A study on the effect of the local bond–slip law characterizing the material bond behavior and its parameters on the global structural bond response of NSM FRP strengthened RC elements has been presented. A numerical method has been developed in order to being able to solve the governing equations of the bonded joint for any type of bond–slip law.

From the comparison between the results of the different bond–slip laws, the following conclusions are obtained:

- In models not including a friction branch after softening, a maximum value of load is attained, which stabilizes for a certain value of the bonded length. In contrast, in models with friction component, the load continuously increases up to a certain slip beyond which only the friction component remains. Furthermore, models with non-linear ascending branch show a stiffener initial load–slip response.
- The non-linear ascending branch effect on the maximum load is practically negligible. Small differences of *P_max_* are observed between BL and TSANL models, and BLF and BONP models, respectively.
- The shape of the bond–slip law has a small effect on the slip profile along the FRP. However, the bond stress and slip distribution at maximum state along the bonded length is strongly affected by the friction branch.
- From the comparison between numerical and experimental results, it can be concluded that:
- A close agreement between the finite differences model and the experimental results is obtained. The comparison between the load–slip curves obtained experimentally and numerically showed that the ascending part is correctly predicted until failure.
- A somewhat larger maximum load of around 7–10% was obtained for specimens with 7.5 mm groove thickness compared to those with 10 mm. As the groove thickness decreased, the maximum load of the bonded joint increased.
- Specimens with 10 mm groove thickness showed a behavior indicating the existence of a friction branch in their local bond law and the failure was in the FRP-adhesive interface. Conversely, the behavior of 7.5 mm groove thickness specimens was properly described using a bilinear function, while the failure was in the resin–concrete interface.
- From the parametric study carried out, the following conclusions can be drawn:
- As the bond–shear strength increases, the maximum load grows, and conversely, the effective bonded length decreases.
- The slip at the bond shear strength, *s*_1_, has a small effect on the maximum load and an increasing effect on the effective bonded length.
- The maximum load and the effective bonded length increase with the maximum slip. Moreover, bond–slip laws without friction branch are much more sensitive to the shifting of the maximum slip.
- The presence of a friction stage in the local bond behavior causes an increase on the maximum load. As the bond–shear strength on the friction branch increases, the maximum load increases as well.

## Figures and Tables

**Table 1:** Equations of the bond–slip models used in the parametric study.

Bond–Slip Model	Equation
Linear Descending (LD) 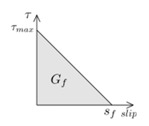	$\tau \left(s\right)=\{\begin{array}{cc} \;\tau_{max}*\frac{\left(s_{f}-s\right)}{s_{f}}, & s>0\;and\;s\leq \;s_{f}\;\\ 0, & s\;\geq \;s_{f} \end{array}$
Bilinear (BL) 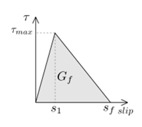	$\tau \left(s\right)=\{\begin{array}{cc} \tau_{max}*\frac{s}{s_{1}}, & s<s_{1}\\ \;\tau_{max}*\frac{\left(s_{f}-s\right)}{\left(s_{f}-s_{1}\right)}, & s>s_{1}\;and\;s\leq \;s_{f}\;\\ \;0, & s\;\geq \;s_{f} \end{array}$
Bilinear-friction (BLF) 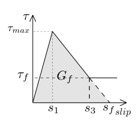	$\tau \left(s\right)=\{\begin{array}{cc} \tau_{max}*\frac{s}{s_{1}}, & s<s_{1}\\ \;\tau_{max}*\frac{\left(s_{f}-s\right)}{\left(s_{f}-s_{1}\right)}, & s>s_{1}\;and\;s\leq \;s_{3}\;\\ \;\tau_{f}, & s\;\geq \;s_{3} \end{array}$
Two-stage ascending non-linear (TSANL) 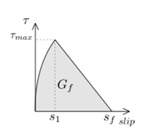	$\tau \left(s\right)=\{\begin{array}{cc} \tau_{max}*{\left(\frac{s}{s_{1}}\right)}^{\propto }, & s<s_{1}\\ \;\tau_{max}*\frac{\left(s_{f}-s\right)}{\left(s_{f}-s_{1}\right)}, & s>s_{1}\;and\;s\leq \;s_{f}\;\\ \;0, & s\;\geq \;s_{f} \end{array}$
Borchert (BO) 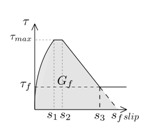	$\tau \left(s\right)=\{\begin{array}{cc} \tau_{max}*{\left(\frac{s}{s_{1}}\right)}^{\propto }, & s<s_{1}\\ \tau_{max}, & s\geq s_{1}\;and\;s\leq s_{2}\\ \;\tau_{max}-\frac{\left(\tau_{max}-\tau_{f}\right)}{\left(s_{f}-s_{1}\right)}*\left(s-s_{1}\right), & s>s_{2}\;and\;s\leq \;s_{3}\;\\ \;\tau_{f}, & s\;\geq \;s_{3} \end{array}$
Borchert no plateau (*s*_1_ = *s*_2_) (BONP) 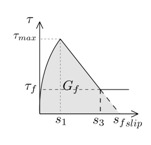	$\tau \left(s\right)=\{\begin{array}{cc} \tau_{max}*{\left(\frac{s}{s_{1}}\right)}^{\propto }, & s<s_{1}\\ \;\tau_{max}-\frac{\left(\tau_{max}-\tau_{f}\right)}{\left(s_{f}-s_{1}\right)}*\left(s-s_{1}\right), & s>s_{1}\;and\;s\leq \;s_{3}\;\\ \;\tau_{f}, & s\;\geq \;s_{3} \end{array}$

Note: τ = bond–shear stress; *s =* slip; *τ_max_* = bond–shear strength; *s*_1_ = slip when the bond–shear strength is achieved; *s*_2_ = final slip of the plateau; *s*_3_ = initial slip of the friction plateau; *s_f_* = maximum slip of the bonded joint; *τ_f_* = friction bond–shear stress; α = experimental parameter that control the shape of the bond–slip law.

**Table 2:** Characteristics of the specimens

Specimen ID	Bonded Length (mm)	Groove Thickness (mm)	Maximum Load (kN)	Failure Mode
NSM – 150 – 10	150	10	43.85	F-A
NSM – 225 – 10	225	10	54.74	F-A
NSM – 150 – 7.5	150	7.5	47.14	C-A
NSM – 225 – 7.5	225	7.5	57.71	C-A

Note: F-A = FRP-adhesive interface, C-A = concrete-adhesive interface.

**Table 3:** Variation of fracture energy for the BONP, BO and BLF models.

		BONP	BO	BLF
***s*** **_1_** **[mm]**	$\frac{s_{1}}{s_{1,0}}$	$\left(\frac{G_{f}-G_{f,0}}{G_{f,0}}\right)*100$	$\left(\frac{G_{f}-G_{f,0}}{G_{f,0}}\right)*100$	$\left(\frac{G_{f}-G_{f,0}}{G_{f,0}}\right)*100$
0.10	1	0 %	0 %	0 %
0.20	2	4.64 %	6.85 %	0 %
0.30	3	9.29 %	13.71 %	0 %
0.40	4	13.94 %	20.56 %	0 %
0.50	5	18.59 %	27.42 %	0 %
